# A Review of the Phenomenon of Hysteresis in the Hypothalamus–Pituitary–Thyroid Axis

**DOI:** 10.3389/fendo.2016.00064

**Published:** 2016-06-14

**Authors:** Melvin Khee-Shing Leow

**Affiliations:** ^1^Division of Medicine, Department of Endocrinology, Tan Tock Seng Hospital, Singapore, Singapore; ^2^Yong Loo Lin School of Medicine, National University of Singapore, Singapore, Singapore; ^3^Brenner Center for Molecular Medicine, Singapore Institute for Clinical Sciences, Singapore, Singapore; ^4^Duke-NUS Medical School, Singapore, Singapore; ^5^Lee Kong Chian School of Medicine, Nanyang Technological University, Singapore, Singapore

**Keywords:** memory effect, lagging TSH recovery, hysteresis, epigenetic regulation, histone modification and chromatin structure

## Abstract

The existence of a phase of prolonged suppression of TSH despite normalization of serum thyroid hormones over a variable period of time during the recovery of thyrotoxicosis has been documented in literature. Conversely, a temporary elevation of TSH despite attainment of euthyroid levels of serum thyroid hormones following extreme hypothyroidism has also been observed. This rate-independent lag time in TSH recovery is an evidence of a “persistent memory” of the history of dysthyroid states the hypothalamus–pituitary–thyroid (HPT) axis has encountered after the thyroid hormone perturbations have faded out, a phenomenon termed “hysteresis.” Notwithstanding its perplexing nature, hysteresis impacts upon the interpretation of thyroid function tests with sufficient regularity that clinicians risk misdiagnosing and implementing erroneous treatment out of ignorance of this aspect of thyrotropic biology. Mathematical modeling of this phenomenon is complicated but may allow the euthyroid set point to be predicted from thyroid function data exhibiting strong hysteresis effects. Such model predictions are potentially useful for clinical management. Although the molecular mechanisms mediating hysteresis remain elusive, epigenetics, such as histone modifications, are probably involved. However, attempts to reverse the process to hasten the resolution of the hysteretic process may not necessarily translate into improved physiology or optimal health benefits. This is not unexpected from teleological considerations, since hysteresis probably represents an adaptive endocrinological response with survival advantages evolutionarily conserved among vertebrates with a HPT system.

## Introduction

Given the exquisite potency of thyroid hormones on the body, the hypothalamus–pituitary–thyroid (HPT) axis is under extremely delicate homeostatic control to ensure that the circulating thyroid hormone levels are finely adjusted to physiological concentrations critical for normal cellular, tissue, and organ development, function as well as the overall survival of the organism (1–3). In human beings, the normal population range of serum-free thyroxine (FT4) lie approximately between 10 and 20 pmol/L, free triiodothyronine (FT3) between 4.0 and 8.0 pmol/L, and that of serum thyrotropin (TSH) between 0.5 and 5.0 mIU/L (4, 5). Within any given individual, there is clear evidence that the normal ranges of the above hormones are much narrower than the population ranges (6–8) and appear to oscillate around a relatively stable and unique mean operating level of FT4 and TSH called the euthyroid set point that defines the individual’s optimal and physiological state of health (9, 10). The HPT axis is naturally regulated by a negative feedback loop in order to keep FT3 and FT4 from swinging off the normal limits. In this system, excessive FT4 (when deiodinated to FT3 intracellularly) and FT3 suppresses the expression of TRH and TSH. Conversely, when FT4 and FT3 are deficient, their lack of inhibition on the hypothalamus and pituitary leads to pronounced elevations of TRH and TSH. Thus, in states of thyroid hormone deficiency when FT3 and FT4 are falling away from the set point and have gone below their lower normal limits, serum TSH will increase and rise beyond the upper limit. During thyroid hormone excess when FT3 and FT4 are rising and have exceeded their upper limits, serum TSH will decline and even become suppressed below the lower limit of normal.

This inverse log-linear pattern of variation between TSH and FT3/FT4 is well known to physiology and medical students as well as doctors and endocrinologists (11, 12). However, a strange observation has been noticed by clinicians treating patients with thyroid hormone disorders. This pertains to elevated serum TSH for a variable period despite restoration of euthyroid levels of FT3/FT4, following treatment of severe hypothyroidism (13, 14). Similarly, it has been noted for decades that serum TSH can become drastically suppressed sometimes for weeks or even months, following the recovery of severe thyrotoxicosis (15, 16). Such a phenomenon of persistent elevation or suppression of serum TSH in the face of normalized FT3/FT4 after recovery of hypo- and hyperthyroidism is termed as “hysteresis” and first described as such in a formal treatise in 2007 (12). Clinicians have been perplexed by this and have also wondered if this implies a residual thyroid dysfunction that deserves treatment to hasten the recovery of serum TSH to the normal range. The following review is devoted to the discussion of hysteresis of the HPT axis and its clinical implications.

## Brief Historical Perspectives of Hysteresis

Hysteresis is a Greek term that means “shortcoming” and “to be late.” It was originally proposed by the late Scottish engineer and physicist, Sir James Alfred Ewing, to refer to the phenomenon observed in systems exhibiting a memory effect such that the response to an input is delayed by a lag time (17). Hysteresis has since been identified in many fields, including physics, economics, and biology. In the area of physiology, hysteresis is encountered in pulmonary mechanics, parathyroid homeostasis, and even urodynamics (18–20). Although the phenomenon of persistent TSH suppression and elevation with consequent lagging of thyrotroph recovery following severe thyrotoxicosis and hypothyroidism had been observed for many years, the first formal description of hysteresis involving the HPT axis was enunciated in 2007 (12).

## Clinical Scenarios

Thyroid function test (TFT) data have revealed that mild departure of FT4 and TSH away from their respective normal ranges often led to the recovery of FT4 and TSH to their expected baseline levels in a coupled fashion fairly rapidly. But when thyroid status swings all the way to the extremes far beyond the limits of the normal ranges, TSH inevitably remained either suppressed or amplified for a variable period of time before finally settling down to the baseline values for any given FT4 or FT3 level. The following illustrates some common examples of TFT disturbances encountered in usual clinical settings.

### Patient 1

The first clinical vignette involves a 35-year-old woman with a history of acute lymphoblastic leukemia as a young child cured with high dose chemoradiation followed by allogeneic bone marrow transplant. She was diagnosed with stage 1 papillary thyroid carcinoma from fine needle aspiration biopsy of a solid 2 cm × 2 cm nodule involving her right thyroid lobe. Pre-surgery TFT revealed a biochemically euthyroid status with serum-free T4 (FT4) level of 14 pmol/L and serum thyrotropin (TSH) level of 2.8 mU/L. Total thyroidectomy was performed, and she was allowed to become hypothyroid before undergoing high dose radioiodine remnant ablation using 100 mCi of I-131. She was then put on TSH-suppressive doses of l-thyroxine (L-T4) till she achieved an FT4 of 21 pmol/L and TSH of 0.09 mU/L. About 2 years later, she was withdrawn from L-T4 for a stimulated thyroglobulin and whole body iodine scanning assessment followed by TSH-suppressive doses of L-T4. The anonymized Table 1 shows her TFT data over time.

**Table 1:** **Change in thyroid function tests of “Patient 1” over time**.

Day	1 (pre-op euthyroid set point values)	14 (total thyroid resection done)	60 (I-131 remnant ablation today)	88 (L-T4 started 2 weeks ago and titrated)	110	290	462	1200	1235	1330
FT4 (pmol/L)	14	8	3	11	16	19	21	4	15	20
TSH (mU/L)	2.8	20.4	66.8	36.3	7.56	0.32	0.09	42.6	24.1	0.15
L-T4 (μg/day)	0	0	0	50	75	112.5	125	0	100	125

**Figure 1:**
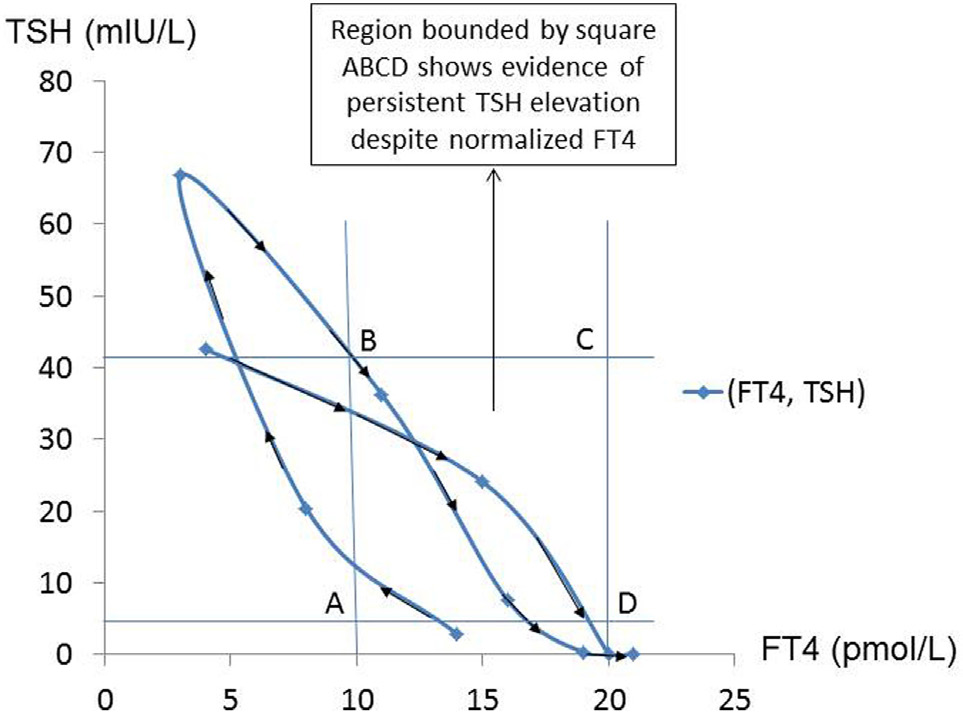
**Arrows trace the trajectory of FT4–TSH, as the patient transited from TSH suppression to persistent TSH elevation, showing hysteresis in operation**.

This can also be illustrated in the form of a graph of TSH vs. FT4 (Figure 1), which revealed the presence of two distinct clockwise hysteresis loops.

### Patient 2

The next clinical vignette involves a typical case of Graves’ disease in a 46-year-old woman. From a TFT done as part of a pre-insurance checkup, her stable euthyroid set point was a FT4 of 15.7 pmol/L and a TSH of 1.25 mIU/L, done when she was 32 years of age. At diagnosis, her FT4 was 57.4 pmol/L, and TSH was suppressed to 0.015 mU/L. She was initiated on carbimazole (CMZ) 30 mg daily till she attained clinical euthyroidism. However, her serum TSH remained persistently suppressed for another 5 months prior to finally becoming biochemically euthyroid after 6 months of antithyroid drug treatment (Table 2).

**Table 2:** **Change in thyroid function tests of “Patient 2” over time**.

Day	Age 32 (euthyroid set point)	Age 46 (diagnosis – Graves’)	Day 42 (CMZ started 6 weeks ago)	84	120	170	215	300	420	600
FT4 (pmol/L)	15.7	57.4	18	16	14	13	10	12	11	14
TSH (mU/L)	1.25	0.015	0.02	0.05	0.16	0.23	2.38	1.60	2.99	1.47
CMZ (mg/day)	0	0	30	20	20	15	10	5	5	2.5

**Figure 2:**
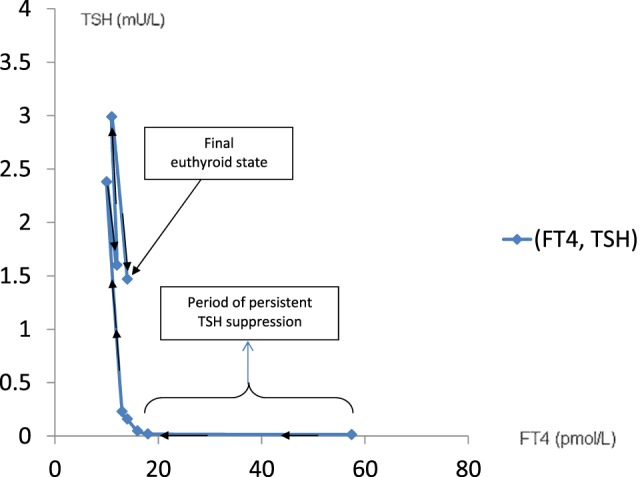
**The course of recovery from hyperthyroidism follows a clockwise hysteresis as illustrated in this graph**. This is typically observed in patients with Graves’ disease treated with antithyroid drugs.

This TFT trajectory circumscribes a clockwise hysteresis, as illustrated below (Figure 2).

### Patient 3

**Figure 3:**
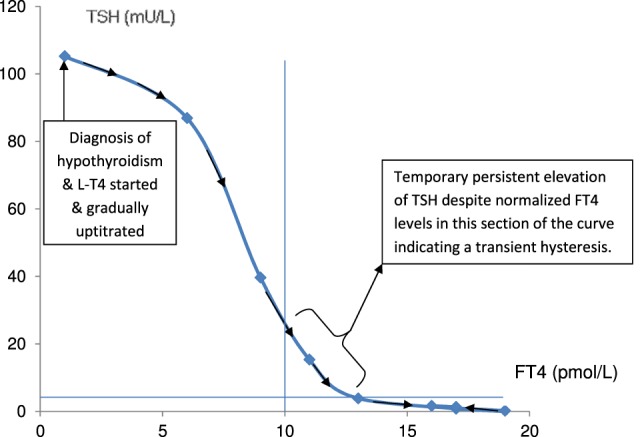
**Graph illustrating the hysteretic path of FT4–TSH in a patient with severe hypothyroidism recovering to a euthyroid state following l-thyroxine replacement**.

The final clinical vignette describes a 54-year-old woman with a strong family history of autoimmune thyroid disease. She presented to the clinic with progressive weight gain, cold intolerance, and constipation. Investigations confirmed Hashimoto’s thyroiditis, and she was put on lifelong L-T4 replacement. She had a normal TFT result taken during a previous medical screen as part of a staff benefit of her employment done about 10 years ago that showed a FT4 of 16 pmol/L and TSH of 1.98 mU/L. The Table 3 shows the TFT trend and the associated graph (Figure 3).

**Table 3:** **Change in thyroid function tests of “Patient 3” over time**.

Day	Age 29 (normal euthyroid set point)	Age 44 (diagnosis: Hashimoto thyroiditis)	Day 14 (L-T4 started 2 weeks ago)	30	90	210	350	450	530	620
FT4 (pmol/L)	16	1	6	9	11	13	19	17	16	17
TSH (mU/L)	1.98	105.21	86.92	39.64	15.33	3.86	0.17	0.79	1.65	1.32
L-T4 (μg/day)	0	0	25	25	50	75	100	87.5	87.5	87.5

## Experimental Evidence of Hysteresis in Lower Vertebrates

**Figure 4:**
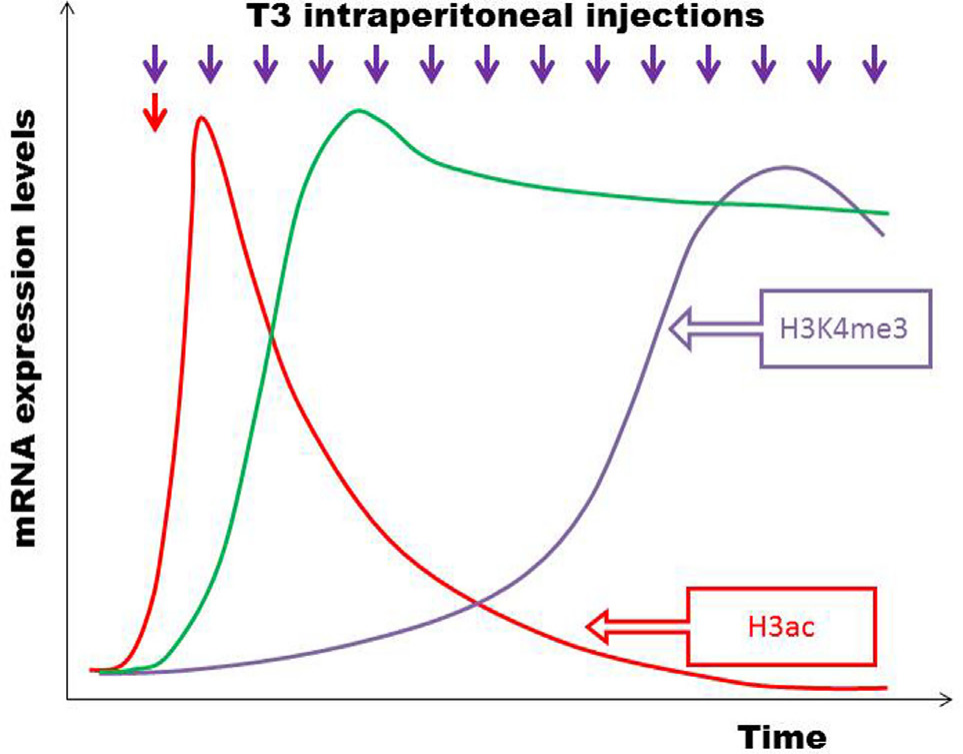
**C57BL/6 mice were divided into two groups – one given a single acute T3 injection (red arrow) and another group given multiple T3 injections intraperitoneally over 14 days (violet arrows)**. Microarrays on liver tissue to study gene expression patterns were done 6 h after acute and chronic T3 injections. Certain target genes responded acutely with a brief increase in mRNA expression, which subsequently became desensitized to T3 and declined (e.g., *Bcl3, Thrsp*) (red line). There were also target genes positively regulated only by chronic T3 exposure, but initially unresponsive to T3 (e.g., *Fgf21*, *Cyp17a1*) (violet line). A third group of genes were positively regulated by both acute and chronic T3 exposure (e.g., *Dio1, Fndc5, Idh3a*) (green line). Different histone modifications influence differential temporal expression patterns during the development of thyrotoxicosis, with H3 acetylation regulating acute T3 responses and H3K4 trimethylation regulating chronic T3 stimulation (21).

**Figure 5:**
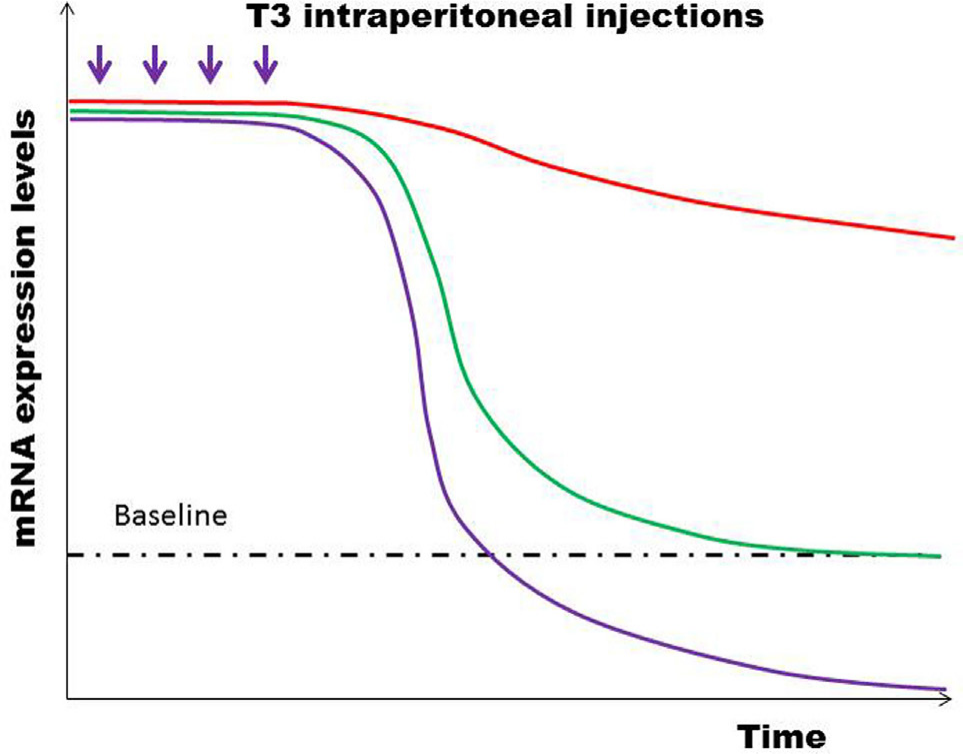
**After the final T3 dose injected, most genes returned rapidly to baseline expression levels (green line), while some genes remained persistently upregulated (red line)**. Still other genes were suppressed below the baseline levels (violet line). Similar patterns of differential temporal expression were also observed in negatively regulated genes (21).

In a study where healthy euthyroid C57BL/6 mice were rendered thyrotoxic with intraperitoneal triiodothyronine (T3), it was observed that serum TSH was suppressed below the normal limit and remained low for a few days despite recovery of serum FT3/FT4 to normal (21). Taking into consideration of the fact that the time scale in small mammalian vertebrates, such as a mouse or rat, is significantly compressed relative to a human being (22–24), this brief period of delayed recovery of TSH is an evidence that the hysteresis phenomenon also occurs in other mammalian species. This demonstrates that hysteresis of the HPT axis occurs most likely through an evolutionarily conserved mechanism and that hysteresis confers a survival advantage (25). Interestingly, it was found that a number of genes were also suppressed to levels below their pre-thyrotoxicosis baseline expression despite normalization of serum T3 and TSH. This implied that thyrotoxicosis is a state that not only leads to a lag time in recovery of TSH but also a delayed recovery of other genes that are regulated by thyroid hormones especially since thyroid hormones regulate an enormous spectrum of genes throughout the body (26, 27). We also discovered that the expression of target thyroid hormone-responsive genes vary according to whether the state of thyroid hormone excess was acute or chronic. Even more interestingly, we have shown for the very first time that epigenetic histone modifications are involved in these differential gene expressions triggered by the thyrotoxic state and that the type of histone mark mediating this was different in acute (H3 acetylation) vs. chronic (H3K4 trimethylation) thyrotoxicosis (Figure 4). Moreover, upon withdrawal of T3 and during the transition from thyrotoxicosis to euthyroidism T3 levels, we showed that about 10% of genes showed incomplete recovery despite normalization of serum T3 and TSH, with some persistently above or below baseline expression (Figure 5). Also, the same pattern was observed among the negatively regulated genes. Hence, at least one of the molecular mechanisms governing the prolonged suppression of the TSH gene is likely to be due to epigenetic histone modifications.

## Modeling

The mathematical modeling of the hysteresis phenomenon is complex and is rarely applied in the field of biology. Hysteresis modeling has a long history dating back to the landmark Preisach paper published in 1935 (28). The Preisach model introduces a hysteresis operator denoted by γ_αβ_ that represents a rectangular input–output loop, where α and β refer to switch in inputs from “up” to “down,” respectively. As an input *u(t)* is monotonically increased, the function proceeds according to an ascending path while the function switches along a descending path distinct from the ascending path when *u(t)* is monotonically decreased. Factoring an arbitrary weighted Preisach function, μ(α,β), this hysteresis operator is given by the double integral as follows:

$$f(t)=\overset{⌢}{\Gamma }u(t)=\iint_{\alpha \geq \beta }\mu (\alpha ,\beta ){\hat{\gamma }}_{\alpha \beta }u(t)d\alpha d\beta$$

where Γ is the Preisach hysteresis operator.

It is instructive to consider various models of HPT axis negative feedback regulation, which can then be modified to include an element of hysteresis as a modeling approach. One such model is exemplified by Pandiyan et al (29). Goede and Leow in 2013 (30) described a simplified hysteresis model of the HPT axis formed by generalization of a negative exponential model. This was based on a clinically validated HPT axis model, represented by this equation (31):

[TSH]=S exp(–φ [FT4])

When remodeled by incorporating a hysteresis factor, ψ, the above model can be expressed as:

[TSH]=S/[ψS+exp(φ [FT4])]

**Figure 6:**
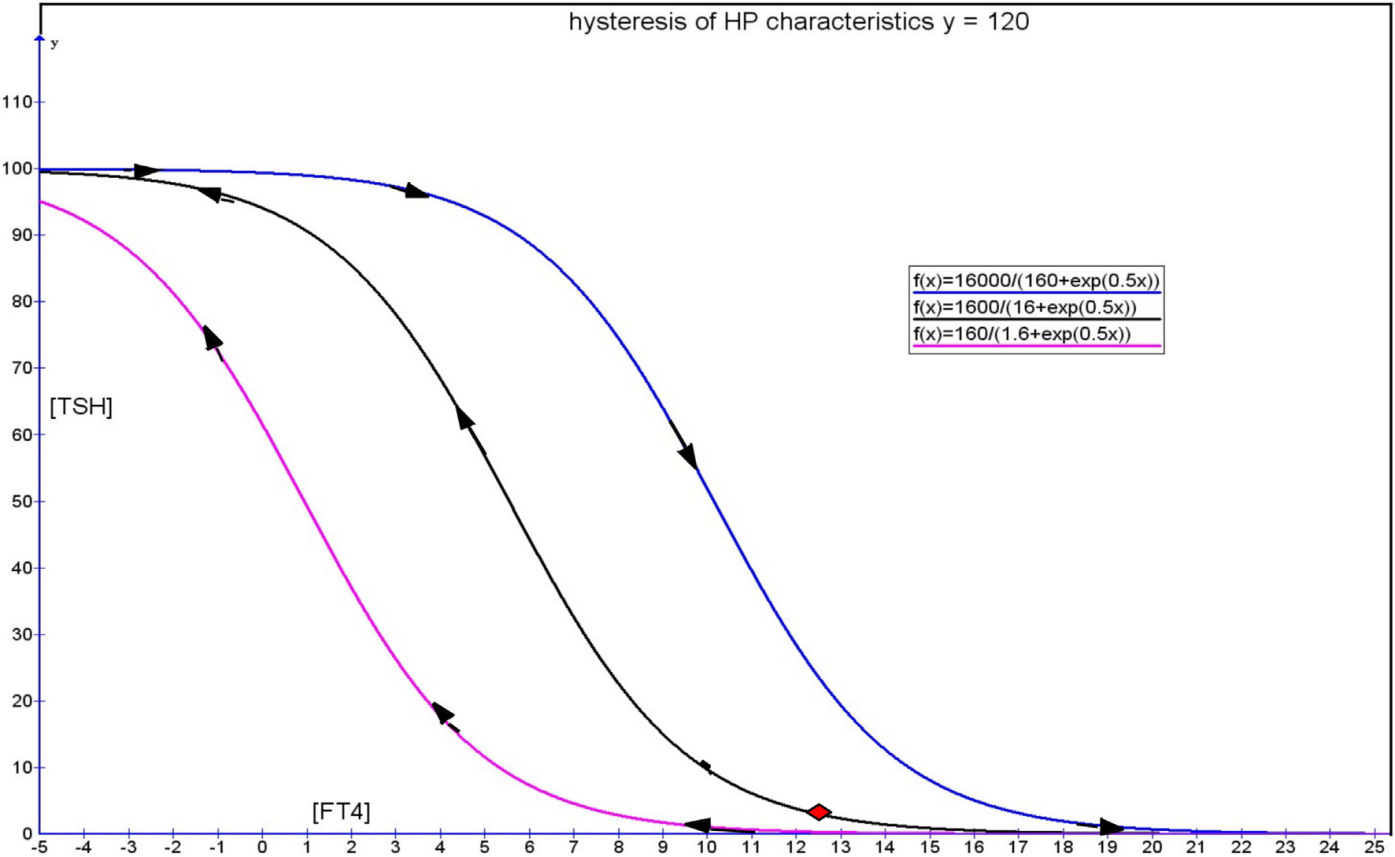
**Hysteresis model showing a clockwise loop formed by an ascending limb represented by the pink curve during recovery of severe thyrotoxicosis and a blue descending limb during recovery from severe hypothyroidism**. The black curve represents the TSH response trajectory that can be followed with more gentle deviations of FT4 from the euthyroid set point indicated by the red diamond. [Figure adapted from Ref. (30)].

This results in saturation effects at the extrema of [FT4] with displacement of the [TSH] function such that the sigmoidal curve is translated horizontally to the left when [FT4] recovers from severe thyrotoxicosis, while the original curve is shifted to the right when [FT4] recovers from severe hypothyroidism. This can be illustrated by the Figure 6. Such a simplified hysteresis model assumes that the maximum TSH response of any individual is known. Obviously, it is difficult in reality to know what the maximum TSH response of any given person is. Based on clinical experience, the [TSH] level of those patients who are severely hypothyroid can range from anywhere between 100 mU/L and well above 400 mU/L or so, giving an idea of the usual maximal magnitude of TSH responses in humans (32). On the contrary, many clinicians have encountered how [TSH] can be suppressed to levels practically close to zero or undetectable (e.g.,<0.005 mU/L) in severe hyperthyroidism. In practice, a realistic value that this hysteresis factor ψ will take that applies to the majority of [TSH] responses is therefore about 0.01. Using this value, it is theoretically possible to deduce what a likely normal euthyroid set point of a patient will be in the absence of hysteresis (i.e., when the effect of hysteresis has fully resolved).

This is understandably a simplified model depicting two hysteresis curves – the forward (recovery from hypothyroidism in blue) and reverse (recovery from hyperthyroidism in red) in a “symmetrical” fashion that are horizontally translated from the original HP curve. In reality, these hysteresis curves are often asymmetric with a more “refractory” reverse hysteresis limb and a relatively more transient forward hysteresis limb. For the purpose of illustration of the concept of hysteresis, a simple model stripped down to the bare essence that contains the elements of delayed recovery to euthyroid TSH levels is shown. This is mainly useful for simulation and teaching purposes. More complex mathematical models of hysteresis will be required to mimic the delayed recovery of TSH for application to individualized patient care. However, such an endeavor is beyond the scope of this article, which is meant to be a brief overarching review of the hysteresis phenomenon in the HPT axis.

## Biological Survival Advantages of Hysteresis

An intriguing question facing physicians is whether the rapid restoration of suppressed or overexpressed [TSH] to normal in thyrotoxic and hypothyroid patients, respectively, who recently achieved normal [FT4] is necessarily a desirable outcome. Occasionally, this poses a concern to anesthesiologists who wondered if it is possible for physicians to normalize [TSH] rapidly in addition to normalizing [FT4] as an optimization of perioperative risk prior to major surgery. Additionally, once [FT4] has been rendered to normal levels but associated with an elevated or suppressed [TSH], it is arguable if this state is pathologically similar to subclinical thyrotoxicosis or subclinical hypothyroidism. Unlike the latter states, what is clear is that the abnormal [TSH] during the recovery of thyrotoxicosis and hypothyroidism is temporary and will ultimately resolve when given time.

Although it may require preclinical and clinical research to elucidate the factors influencing the duration to recovery from prolonged elevated or suppressed [TSH], an important clue comes from questioning the reason why nature has engineered such a response to cope with swings in hormones with great potency. Thyroid hormone belongs to this category in which adequate levels are critical to survival and yet life threatening when excessive or deficient. Is it any wonder then that [TSH] should remain suppressed for weeks that sometimes dragged to months or years in someone suffering from severe hyperthyroidism whose [FT4] has been brought down effectively by antithyroid drugs? When one analyzes this situation, it becomes apparent that the hysteresis with a lagging recovery in [TSH] helps to protect the individual from accelerated rebound hyperthyroidism in case antithyroid drugs are suddenly discontinued prematurely for whatever reason because the persistent suppressed [TSH] implies negligible TSH stimulation on the unrestrained overactive thyroid. Had [TSH] been normalized rapidly following severe hyperthyroidism, then sudden cessation of antithyroid drugs leading to rapid escalation of [FT4] will be compounded further by extra TSH stimulation on the thyroid follicles to generate even greater [FT4]. In the same vein, for an individual who requires l-thyroxine replacement for severe primary hypothyroidism, the prolonged elevation in [TSH] meant that there is an attempt by the body to continue maximally stimulating the thyroid, in case l-thyroxine should be unexpectedly stopped.

**Figure 7:**
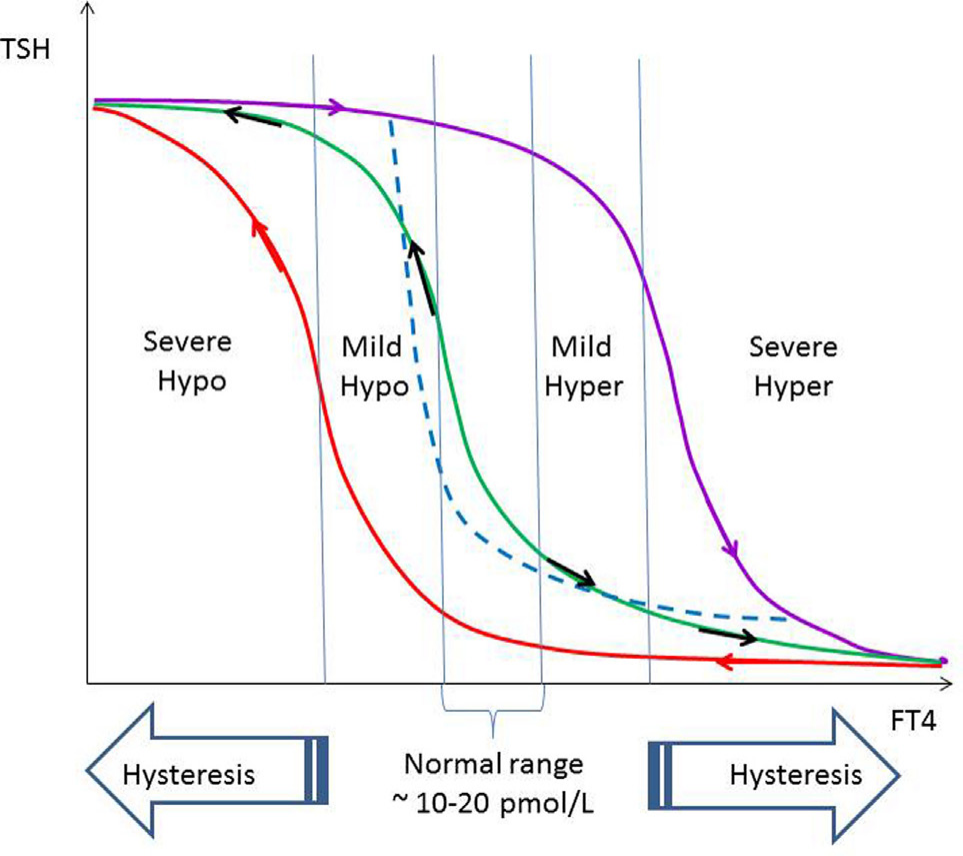
**The relationship of FT4 and TSH is a negative exponential function with saturation characteristic, as shown by the green curve (12)**. Within the normal range of FT4 and slightly beyond on either sides denoted by mild hypothyroidism and hyperthyroidism, respectively, recovery of TSH is more rapid and not subjected to hysteresis. The FT4–TSH relationship within the non-hysteresis boundaries may be approximated by the dashed blue negative exponential curve without saturation (10). In severe hypothyroidism and hyperthyroidism, recovery of TSH lags behind recovery of FT4 significantly due to hysteresis (violet and red lines, respectively).

Therefore, hysteresis of the HPT axis serves as a buffering mechanism to reduce the magnitude of the biological impact of severe hyperthyroidism or hypothyroidism on the organism, especially when thyroid hormones escalate to extreme levels at either side of the normal (Figure 7). While speculative, this buffering capacity offered by the hysteresis phenomenon probably confer a survival advantage and is thus expected to be evolutionarily conserved among all vertebrate species depending on a thyroid system for development, metabolism, and survival. Although the TSH gene is the focus of this treatise on the hysteresis of the HPT axis, it is likely that the multitude of other crucial genes governed by thyroid hormones are also potentially subjected to this hysteresis phenomenon and may thus take a variable period of time to return back to baseline following severe hypothyroidism or hyperthyroidism.

## Clinically Relevant Considerations and Applications

Although suppressed or elevated [TSH] in a real situation of hysteresis is often obvious, it is important to consider the possibility that TSH abnormalities can occasionally be the result of drug or antibody interferences with assay platforms (33–35). In this respect, the presence of certain heterophile antibodies, such as human anti-mouse monoclonal antibodies (HAMA), can lead to artifactual elevations [TSH] depending on the reactivity of these heterophile antibodies with the detection antibodies in the assay systems. Patients taking biotin supplements can also face the issue of falsely elevated or suppressed [TSH] in biotin-streptavidin affinity-based assays (36). In addition, the prolonged suppression of TSH itself may be contributed by the regulation of TSH secretion *via* ultrashort autocrine loop at the hypothalamic–pituitary level, as supported by the expression of TSH receptors in the folliculo-stellate cells in the anterior pituitary (37–39). Rate-dependent “hysteresis” due to a dynamic lag between input and output such as turnover kinetics of hypothalamic TRH biosynthetic enzymes coupled with varying degrees of enzymatic induction or repression in response to signals establishing new homeostatic equilibria may potentially contribute to the overall observed hysteresis as well. Finally, there is a theoretical possibility among those on l-thyroxine (L-T4) replacement that the timing of blood sampling relative to their L-T4 dosing may pose a confounding suppressive effect on [TSH], as an increase of up to 14% in [FT4] would be expected if L-T4 was ingested prior to blood sampling, assuming a half-life of 7 days for [FT4]. In practice, this is probably insignificant, which means patients need not withhold L-T4 prior to the blood draw as TSH secretion rate does not respond so quickly to such degrees of changes in ambient [FT4] (40, 41).

## Conclusion

The relationship of [FT4] and [TSH] is a reciprocal one best described by a negative exponential model. Hyperthyroidism and hypothyroidism lead to temporary suppression and overexpression of TSH out of the normal reference range. Even fluctuations of [FT4] within its normal reference range are associated with perceptible reciprocal changes in [TSH]. Mild displacements of [FT4] off the normal limits seldom result in any lagged recovery in TSH. However, in more extreme cases of hyperthyroidism or hypothyroidism, TSH is often appropriately suppressed or overexpressed for a protracted period of time despite adequate treatment that renders [FT4] into the normal range. This phenomenon is now recognized as hysteresis of the HPT axis and probably represents an adaptive response that confers a biological survival advantage for the organism. Hence, HPT axis hysteresis may be evolutionarily conserved and could well operate in vertebrates other than humans, as has been demonstrated in a mouse model. The implication of hysteresis acting as protective buffer may imply that rapid restoration of [TSH] to normal during this lagging recovery phase is not necessarily desirable or advantageous in terms of optimization of the euthyroid state compared to recovery of [TSH] along a slower trajectory.

## Author Contributions

ML conceived this work, drafted the manuscript, critically reviewed its intellectual content, gave final approval of the version to be published, and agreed to be accountable for all aspects of the work in ensuring that questions related to the accuracy or integrity of any part of the work are appropriately investigated and resolved.

## Conflict of Interest Statement

Exploit Technologies Pte Ltd. (ETPL), A*STAR’s tech-transfer arm, has filed a patent on the HPT axis set point algorithm that has been developed into a computer program (Thyroid-SPOT software), and ML is listed as one of the three coinventors. The patent is successfully granted in Singapore.
